# The impacts of hypertonic conditions on *Drosophila* larval cool cells

**DOI:** 10.3389/fncel.2024.1347460

**Published:** 2024-09-23

**Authors:** Hua Bai, Trisha Naidu, James B. Anderson, Hector Montemayor, Camie Do, Lina Ni

**Affiliations:** School of Neuroscience, Virginia Polytechnic Institute and State University, Blacksburg, VA, United States

**Keywords:** *Drosophila* larvae, dorsal organ cool cells, ionotropic receptors, temperature responses, hypertonicity

## Abstract

*Drosophila melanogaster* exhibits multiple highly sophisticated temperature-sensing systems, enabling its effective response and navigation to temperature changes. Previous research has identified three dorsal organ cool cells (DOCCs) in fly larvae, consisting of two A-type and one B-type cell with distinct calcium dynamics. When subjected to hypertonic conditions, calcium imaging shows that A-type DOCCs maintain their responses to cool temperatures. In contrast, a subset of B-type DOCCs does not exhibit detectable GCaMP baseline signals, and the remaining detectable B-type DOCCs exhibit reduced temperature responses. The activation of both A-type and B-type DOCCs depends on the same members of the ionotropic receptor (IR) family: IR21a, IR93a, and IR25a. A-type DOCCs exhibit a higher somal level of IR93a than B-type DOCCs. Overexpression of *Ir93a* restores B-type calcium responses to cool temperatures, but not the proportion of B-type cells with a detectable GCaMP baseline, in a hypertonic environment, suggesting a selective role of IR93a in maintaining the temperature responses under hypertonic conditions. Our findings identify a novel function of B-type DOCCs in integrating temperature and tonic stimuli.

## Introduction

Temperature is a universal variable that humans and all other living organisms consistently encounter. These organisms depend on their thermosensory systems to detect the constantly changing environmental temperatures, guiding them to avoid potentially dangerous temperature extremes and seek out optimal conditions for their survival and reproduction (Morrison and Nakamura, 2019; Xiao and Xu, 2021; Franks and Hoffmann, 2012). Temperature variation is vital for small ectotherms such as fruit flies, which rely on ambient temperatures to set their body temperatures (Garrity et al., 2010; Dillon et al., 2010). Both adult flies and larvae possess multiple thermosensory systems activated by different temperature ranges (Xiao and Xu, 2021; Li and Gong, 2017; Barbagallo and Garrity, 2015).

**FIGURE 1:**
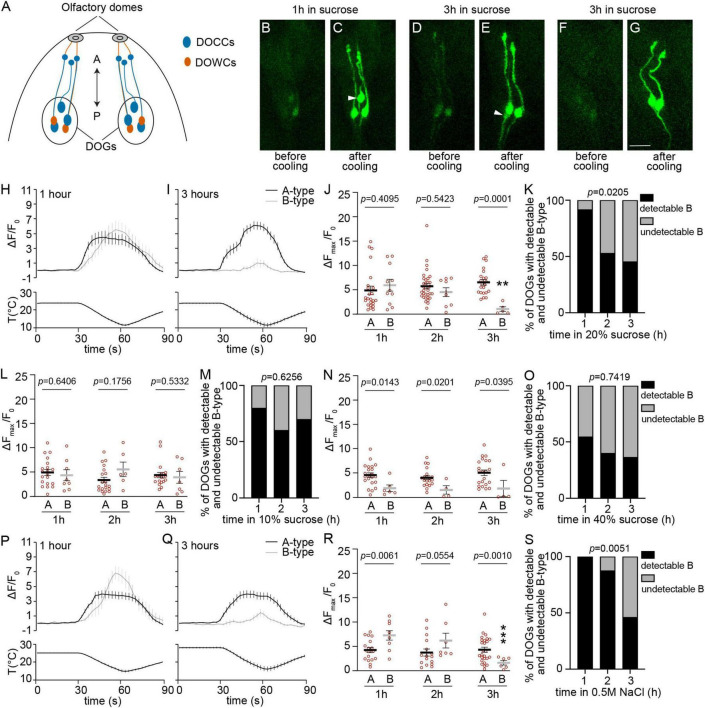
The temperature responses of detectable B-type DOCCs are not maintained under hypertonic conditions. **(A)** The anterior part of a fly larva is depicted. Each dorsal organ ganglion (DOG) contains three DOCCs (blue ovals) and two DOWCs (orange ovals). Each DOCC possesses a “dendrite bulb” (blue circles), while DOWC dendrites extend to olfactory domes. The double-headed arrow indicates the anterior-posterior axis. **(B–G)** Temperature responses of DOCCs labeled by *Ir21a-Gal4/UAS-GCaMP6m* with the exposure to 20% sucrose for either up to one hour **(B,C)** or between two and three hours **(D–G)**. White arrowheads denote B-type DOCCs. Scale bars: 10 μm. **(H,I)** Calcium changes in response to temperature fluctuations of indicated DOCCs and durations in 20% sucrose. Fluorescence is quantified as a ratio of fluorescence intensity at the indicated time points to initial intensity. **(J)** The ratio of maximum fluorescence to initial fluorescence of indicated DOCCs and durations in 20% sucrose. Mann–Whitney test or unpaired *t*-test, *p*-values are displayed in the figure. **p < 0.01, Welch’s *t*-test for comparing B-type responses following exposure to 20% sucrose for one and three hours. 1 h: 24 A-type DOCCs and 11 B-type DOCCs from 12 DOGs; 2 h: 34 A-type DOCCs and 9 B-type DOCCs from 17 DOGs; 3 h: 22 A-type DOCCs and 5 B-type DOCCs from 11 DOGs. **(K)** The percentage of DOGs containing detectable B-type DOCCs decreases as the duration of 20% sucrose exposure increases. Chi-square test for trend, *p*-value is displayed in the figure. **(L)** The ratio of maximum fluorescence to initial fluorescence of indicated DOCCs and durations in 10% sucrose. Mann–Whitney test or unpaired *t*-test, *p*-values are displayed in the figure. 1 h: 20 A-type DOCCs and 8 B-type DOCCs from 10 DOGs; 2 h: 20 A-type DOCCs and 6 B-type DOCCs from 10 DOGs; 3 h: 20 A-type DOCCs and 7 B-type DOCCs from 10 DOGs. **(M)** The percentage of DOGs containing detectable B-type DOCCs of the indicated durations of 10% sucrose exposure. Chi-square test for trend, *p*-value is displayed in the figure. **(N)** The ratio of maximum fluorescence to initial fluorescence of indicated DOCCs and durations in 40% sucrose. Mann–Whitney test, *p*-values are displayed in the figure. 1 h: 23 A-type DOCCs and 6 B-type DOCCs from 13 DOGs; 2 h: 20 A-type DOCCs and 4 B-type DOCCs from 10 DOGs; 3 h: 22 A-type DOCCs and 4 B-type DOCCs from 11 DOGs. **(O)** The percentage of DOGs containing detectable B-type DOCCs of the indicated durations of 40% sucrose exposure. Chi-square test for trend, *p*-value is displayed in the figure. **(P,Q)** Calcium changes in response to temperature fluctuations of indicated DOCCs and durations in 0.5 M NaCl. Fluorescence is quantified as a ratio of fluorescence intensity at the indicated time points to initial intensity. **(R)** The ratio of maximum fluorescence to initial fluorescence of indicated DOCCs and durations in 0.5 M NaCl. Mann–Whitney test or unpaired *t*-test, *p*-values are displayed in the figure. ***p < 0.001, unpaired *t*-test for comparing B-type responses following exposure to NaCl for one and three hours. 1 h: 18 A-type DOCCs and 9 B-type DOCCs from 9 DOGs; 2 h: 16 A-type DOCCs and 7 B-type DOCCs from 8 DOGs; 3 h: 26 A-type DOCCs and 6 B-type DOCCs from 13 DOGs. **(S)** The percentage of DOGs containing detectable B-type DOCCs of the indicated durations of NaCl exposure. Chi-square test for trend, *p*-value is displayed in the figure. Data represent mean ± s.e.m.

In fly larvae, each dorsal organ ganglion (DOG) houses three cool-activated cells (DOCCs) (Figure 1A). The activation of DOCCs depends on three members of the ionotropic receptor (IR) family: IR21a, IR25a, and IR93a (Ni et al., 2016; Knecht et al., 2016). The IR family is a branch of the ionotropic glutamate receptor (iGluR) family, which is predominantly found in sensory neurons and mediates diverse sensory transductions in flies and other invertebrates (Ni, 2020). Like iGluRs, four IR subunits form a heteromeric complex (Abuin et al., 2019; Rytz et al., 2013). DOCCs and their temperature-sensitive IRs are necessary for thermotactic navigation when animals are exposed to a shallow temperature gradient (Klein et al., 2015; Hernandez-Nunez et al., 2021; Tyrrell et al., 2021). While optogenetic experiments demonstrate that DOCCs drive avoidance of stimuli, they also contribute to cool temperature attractive behaviors, such as guiding animals to leave 25°C and move toward 18°C in response to a sudden temperature increase from 18 to 25°C (Klein et al., 2015; Hernandez-Nunez et al., 2021; Tyrrell et al., 2021; Omelchenko et al., 2022b).

Although molecular thermoreceptors in both A-type and B-type cells consist of the same three IRs, they respond differently to cool temperatures and thus are divided into two types. In each DOG, there are two A-type DOCCs and one B-type DOCC. When animals are subjected to negative linear temperature ramps from 33 to 15°C, A-type and B-type DOCCs are activated at about 32°C and 26°C, respectively (Klein et al., 2015). Morphologically, A-type DOCCs pair with warm cells (DOWCs) expressing IR25a, IR93a, and IR68a (Figure 1A), while B-type DOCCs pair with an unidentified cell (Hernandez-Nunez et al., 2021). However, the mechanism underlying these distinct responses between A-type and B-type DOCCs is still unknown.

Tonicity is the ability of a solution to drive water movement across the cell membrane by osmosis (Maldonado and Mohiuddin, 2023). Temperature and tonicity are closely related environmental factors. Temperature affects the rate of osmosis, which in turn impacts tonicity. Animals such as *C. elegans* depend on the same molecular complex, OSM-9 and OCR-2, to respond to temperature and tonic stimuli (Colbert et al., 1997; Bargmann et al., 1990; Tobin et al., 2002; Ohnishi et al., 2020). In this study, we characterized the discrepancies between A-type and B-type DOCCs by subjecting animals to hypertonic conditions. Calcium imaging demonstrated that *wild-type* (*wt*) A-type DOCCs remained unaffected by hypertonic conditions. In contrast, the proportion of B-type DOCCs with a detectable GCaMP baseline was reduced, and the temperature responses of the remaining detectable B-type DOCCs declined, suggesting B-type DOCCs may function as integrators for thermal and tonic information. Immunohistochemistry analysis revealed that the somal expression of IR93a was significantly higher in A-type DOCCs than in B-type DOCCs. Overexpression of *Ir93a* increased the IR93a somal level in B-type DOCCs, which restored the calcium responses of detectable B-type DOCCs under hypertonic conditions. However, the proportion of B-type DOCCs exhibiting detectable GCaMP baseline signals was not restored, indicating that different mechanisms regulate the detectability and responses of B-type DOCCs in a hypertonic environment, and that the IR93a level impacts their temperature responses but not detectability.

## Materials and methods

### Fly strains

*Canton-S* (*CS*) was used as the *wild type* (*wt*) control. The following flies were previously described: *Ir21a-Gal4* (Ni et al., 2016), *Ir68a-Gal4* (Knecht et al., 2017), *Ir21a^Δ1^* (Ni et al., 2016), *Ir25a*^2^ (Benton et al., 2009), *Ir93a^Mi^* (Knecht et al., 2016), *UAS-Ir21a* (Ni et al., 2016), *UAS-Ir25a* (Abuin et al., 2011), *UAS-Ir93a* (Knecht et al., 2016), *UAS-GFP* [*p(10XUAS-IVS-Syn21-GFP-p10)attP2*] (Pfeiffer et al., 2012), *UAS-TrpA1* (Hamada et al., 2008), and *UAS-GCaMP6m* [*p(20XUAS-IVS-GCaMP6m)attP40* and *p(20XUAS-IVS-GCaMP6m)VK00005*] (RRID:BDSC_42748 and BDSC_42750) (Chen et al., 2013).

### Calcium imaging

Calcium imaging and analysis of larval neurons to temperature changes were performed as described (Tyrrell et al., 2021; Omelchenko et al., 2022a; Omelchenko et al., 2022b). Fly larvae at 72 h AEL were collected using 10 mL 20% w/v sucrose solution. The duration in 20% sucrose solutions was counted from the moment that the 20% sucrose solution was added to the fly vial. Approximately 15–20 min later, larvae were transferred into 100 μL drops of 20% sucrose; each drop contained 20–30 larvae. If other hypertonic solutions (10% sucrose, 40% sucrose, and 0.5 M NaCl) were applied, larvae were picked using a small paint brush and then placed in the hypertonic solutions. The duration in hypertonic solutions was counted from the moment that larvae were placed in the hypertonic solutions. When calcium imaging was conducted, a 3rd-instar larva was selected using a stereo microscope and transferred from hypertonic solutions to 75 μL of 1× PBS on a slide for imaging, and the time the larva spent in the hypertonic solutions was recorded. We combined several criteria to distinguish between A-type and B-type DOCCs. Within a DOG, the B-type DOCC is the most anterior DOCC and tends to have shorter dendrites than A-type DOCCs (Hernandez-Nunez et al., 2021). In addition, A-type DOCCs also typically exhibit quicker responses than B-type DOCCs, and the calcium changes in two A-type cells generally occur at concurrent or similar time points (Klein et al., 2015).

Calcium imaging was conducted on a Zeiss LSM 880 using the Airyscan Fast mode, equipped with a *z*-axis piezo stage (432339-9000-000, Wienecke & Sinske) and a stage insert (432339-9030-000, Wienecke & Sinske). Z-stacks were captured at 7–8 fps with a 512 × 512 resolution and 1.5× zoom using a 25× water objective. A custom-built thermoelectric cooler was made to deliver the temperature stimuli by attaching a thermoelectric module (30 mm × 30 mm, TE-127-1.0–0.8, TE Technology) to a modified heat sink (12.9 cm × 5.5 cm, ATS2193-ND, Digi-Key). The cooler was placed on the slide covering the larvae, and a 2A current was supplied using a power supply (CSI1802X, Circuit Specialists). The temperature was monitored using a data acquisition device (USB-TEMP, Measurement Computing) and DAQami software (Measurement Computing). Images were analyzed using ImageJ plugins TrackMate and TACI (Tinevez et al., 2017; Omelchenko et al., 2022a).

### Immunostaining

Immunostaining was performed as described (Kang et al., 2011). The following antibodies were used: guinea pig anti-IR21a (1:100) (Budelli et al., 2019), rabbit anti-IR93a (1:100) (Knecht et al., 2016), guinea pig anti-IR25a (1:100) (Benton et al., 2009), chicken anti-GFP (1:500; Abcam), goat anti-guinea pig Cyanine Cy™3 (1:100; Jackson ImmunoResearch), goat anti-rabbit Alexa Fluor^®^ 647 (1:100; Jackson ImmunoResearch), and goat anti-chicken FITC (1:500; Invitrogen). IR21a, IR93a, and IR25a antibodies were kind gifts from Dr. Richard Benton. To quantify immunostaining, the center plane of each soma was determined in ImageJ. The mean intensity was then read (Analyze > Measure) and the background intensity was subtracted.

### Statistical analysis

Statistical details of experiments are described in figure legends. The normality of distributions was assessed by the Shapiro–Wilk test (*p* ≤ 0.05 rejected normal distribution). Statistical comparisons of normally distributed data were performed by the one sample *t*-test, the two-tailed unpaired *t*-test, the Welch’s *t*-test, or the ordinary one-way ANOVA test followed by the Tukey test. For data that did not conform to a normal distribution, statistical comparisons were performed by the one sample Wilcoxon test, the Mann–Whitney test, or the Kruskal–Wallis test followed by the Dunn’s test. Chi-square test for trend or Fisher’s exact test was used in analyzing the detectability of DOCCs. Data analysis was performed using GraphPad Prism 9.

## Results

### The temperature responses of B-type DOCCs are not maintained under hypertonic conditions

We first tested the temperature responses of DOCCs in 20% sucrose using calcium imaging. The duration of sucrose exposure affected the temperature responses of B-type DOCCs. When larvae stayed in the sucrose solution for no more than one hour, most animals exhibited detectable GCaMP baseline signals in B-type DOCCs with robust calcium responses to cool temperatures, similar to A-type responses (Figures 1B, C, H, J, K). However, when the duration of exposure to sucrose was extended to three hours, more than half of the animals no longer exhibited detectable B-type DOCCs (Figures 1F, G, K). The undetectability of B-type DOCCs was not due to the absence of B-type DOCCs but because their GCaMP signals fell below the detection threshold for two reasons. First, B-type DOCCs remained labeled by IR21a, IR25a, and IR93a antibodies under hypertonic conditions (Supplementary Figure 1A). Second, B-type DOCCs that were undetectable at baseline exhibited warm-activated responses when expressing a warm-activated temperature receptor TRPA1 (Supplementary Figure 2). For the B-type DOCCs that were detectable by calcium imaging, their temperature responses significantly decreased and were smaller than those of A-type DOCCs (Figures 1D, E, I, J). Sucrose exposure did not decrease the cooling responses or the baseline detectability of A-type DOCCs (Figures 1B–J).

We then tested the dose effect using different concentrations of sucrose solutions. When larvae were exposed to 10% sucrose for three hours, we did not observe a reduction in the proportion of detectable B-type cells or their temperature responses (Figures 1L, M). This result is as expected because 10% sucrose is a relatively weaker hypertonic solution and, thus, does not exert a significant effect within the tested time frames. In contrast, exposure to 40% sucrose influenced the responses and detectability of B-type DOCCs within the first hour of incubation (Figures 1N, O). One hour of incubation in 40% sucrose led to similar effects on B-type cells to those exposed to 20% sucrose for three hours: neither the temperature responses in detectable B-type cells (Mann–Whitney test, *p* = 0.4286) nor the detectability (Fisher’s exact test, *p* > 0.9999) showed a significant difference between these two conditions. We did not observe a further decrease in the proportion of B-type DOCCs with a detectable GCaMP baseline following prolonged exposure to 40% sucrose (Figure 1O). In addition, the responses of detectable B-type DOCCs did not further diminish (Figure 1N). This indicate that one-hour incubation in 40% sucrose has already exerted the maximum impact on B-type DOCCs, and thus no further decrease in detectability or responses can be observed.

In addition to sucrose, we tested another hypertonic solution 0.5 M NaCl. Similar to 20% sucrose, 0.5 M NaCl had no effect on A-type DOCCs after three hours of incubation (Figures 1P–R). However, the responses of detectable B-type DOCCs significantly declined (Figures 1P–R). Furthermore, the detectability of B-type DOCCs also decreased (Figure 1S). These findings suggest hypertonic environments selectively modulate B-type DOCCs.

### B-type DOCCs express a lower somal level of IR93a than A-type DOCCs

**FIGURE 2:**
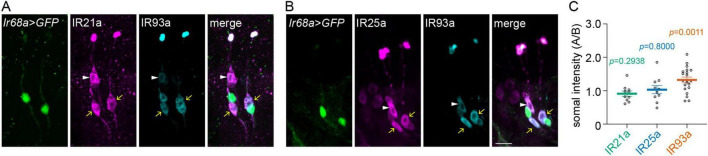
A-type DOCCs express a higher level of IR93a than B-type DOCCs. IR25a and IR93a are expressed in both DOCCs and DOWCs. IR21a is expressed in DOCCs, while *Ir68a-Gal4* is expressed in DOWCs. **(A,B)** Immunostaining of GFP [green; *Ir68a* > *GFP* (*Ir68a-Gal4/UAS-GFP*)], IR21a (magenta in A), IR25a [magenta in **(B)**], and IR93a (cyan) in the anterior part of fly larvae. White arrowheads denote B-type DOCCs, and yellow arrows denote A-type DOCCs. Scale bar: 10 μm. **(C)** The somal fluorescence intensities of IR21a, IR25a, and IR93a in A-type to B-type DOCCs. The intensities of two A-type DOCCs within the same DOG were averaged, followed by the calculation of the intensity ratios of A-type over B-type DOCCs. One sample *t*-test, *p*-values are displayed in the figure. Data represent mean ± s.e.m. IR21a: 20 A-type and 10 B-type DOCCs from 5 animals; IR25a: 20 A-type and 10 B-type DOCCs from 5 animals; IR93a: 40 A-type and 20 B-type DOCCs from 10 animals.

To explore the mechanisms that underlie the discrepancy in cooling responses between A-type and B-type DOCCs under hypertonic conditions, we examined the expression levels of IRs (IR21a, IR25a, and IR93a) in the soma. The specificity of IR antibodies was validated using the corresponding *Ir* mutants (Supplementary Figure 3). A-type DOCCs (yellow arrows) paired with DOWCs, labeled by *Ir68a* > *GFP* (Figures 2A, B). B-type DOCCs (white arrowheads) paired with an IR25a-positive cell that did not express IR21a, IR93a, or *Ir68a-Gal4* (Figures 2A, B). When we quantified the somal expression of each IR, we observed that IR93a expression was significantly higher in A-type than B-type DOCCs, while IR21a and IR25a exhibited similar levels in both (Figure 2C and Supplementary Table 1). Hypertonic conditions had no impact on the expression patterns of IRs (Supplementary Figure 1A and Supplementary Table 1). These results suggest that the levels of IR subunits may play a role in determining the different cooling responses of A-type and B-type DOCCs under hypertonic conditions.

### Overexpression of *Ir25a* and *Ir93a* impacts the cooling responses of DOCCs

**FIGURE 3:**
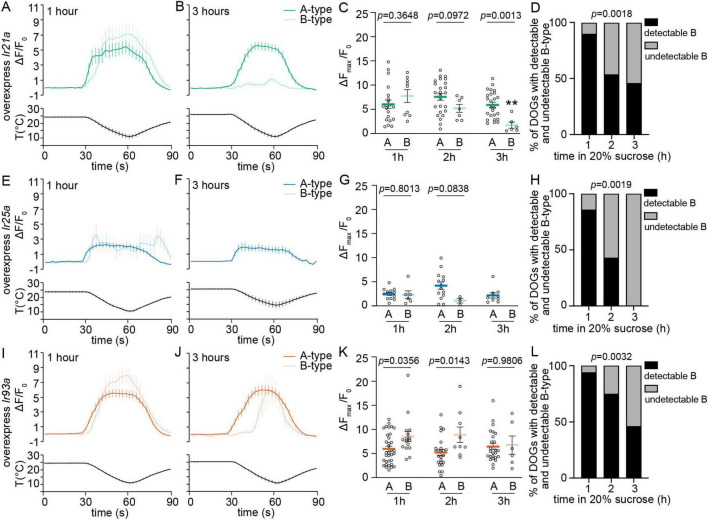
Overexpression of *Ir93a* rescues the temperature responses of detectable B-type DOCCs, not the detectability of B-type DOCCs, with prolonged exposure to hypertonic solutions. **(A,B,E,F,I,J)** Calcium changes in response to temperature fluctuations of indicated genotypes, cell types, and durations in 20% sucrose. Fluorescence is quantified as a ratio of fluorescence intensity at the indicated time points to initial intensity. **(C,G,K)** The ratios of maximum fluorescence to initial fluorescence of indicated genotypes, cell types, and durations in 20% sucrose. Unpaired *t*-test or Mann–Whitney test, *p*-values are displayed in the figure. **p < 0.01, Welch’s *t*-test for comparing B-type responses following exposure to 20% sucrose for one and three hours. **(C)** 1 h: 20 A-type DOCCs and 9 B-type DOCCs from 10 DOGs; 2 h: 25 A-type DOCCs and 7 B-type DOCCs from 13 DOGs; 3 h: 25 A-type DOCCs and 6 B-type DOCCs from 13 DOGs. **(G)** 1 h: 13 A-type DOCCs and 6 B-type DOCCs from 7 DOGs; 2 h: 14 A-type DOCCs and 3 B-type DOCCs from 7 DOGs; 3 h: 9 A-type DOCCs and 0 B-type DOCCs from 5 DOGs. **(K)** 1 h: 34 A-type DOCCs and 16 B-type DOCCs from 17 DOGs; 2 h: 24 A-type DOCCs and 9 B-type DOCCs from 12 DOGs; 3 h: 25 A-type DOCCs and 6 B-type DOCCs from 13 DOGs. **(D,H,L)** The percentage of DOGs containing detectable B-type DOCCs of indicated genotypes and durations in 20% sucrose. Chi-square test for trend, *p*-values are displayed in the figure. Data represent mean ± s.e.m. Genotype: overexpress *Ir21a*: *Ir21a-Gal4/+;UAS-GCaMP6m/UAS-Ir21a*; overexpress *Ir25a*: *Ir21a-Gal4/UAS-Ir25a;UAS-GCaMP6m/+*; overexpress *Ir93a*: *Ir21a-Gal4/+;UAS-GCaMP6m/UAS-Ir93a*.

To explore whether the discrepancies in the detectability and temperature responses between A-type and B-type DOCCs were due, at least in part, to the different levels of IRs, we expressed *UAS-Ir21a*, *UAS-Ir25a*, and *UAS-Ir93a* in both A-type and B-type DOCCs using *Ir21a-Gal4*. Overexpressing *Ir21a* did not impact calcium responses of detectable B-type DOCCs (Figures 3A–C) or the proportion of animals with detectable B-type DOCCs compared to *wt* (Figure 3D). Therefore, overexpression of *Ir21a* did not alter B-type responses with or without prolonged hypertonic exposure.

Overexpression of *Ir25a* had a noticeable impact on the responses and detectability of both A-type and B-type DOCCs. When *Ir25a* was overexpressed, more than half of the animals failed to display detectable A-type DOCCs, but the proportion of animals with detectable A-type DOCCs did not decline with prolonged exposure to sucrose (Supplementary Figure 4A). Moreover, the responses of A-type DOCCs remained unchanged over time in sucrose (Figures 3E–G). We could not observe B-type DOCCs in animals that did not exhibit detectable A-type DOCCs. Unlike A-type DOCCs, the proportion of animals with detectable B-type DOCCs decreased with extended exposure to sucrose (Supplementary Figure 4B). When considering only animals with detectable A-type DOCCs, the proportion of animals exhibiting detectable B-type DOCCs resembled that of *wt* animals (Figure 3H). Like in *wt*, the responses of detectable B-type DOCCs diminished over time in sucrose. After three hours, no detectable B-type DOCCs were observed (Figures 3E–G). Therefore, the overexpression of *Ir25a* did not change how hypertonic conditions impacted their responses, even though it led to a reduction in the proportion of animals with detectable A-type and B-type DOCCs and their responses without extended exposure to hypertonic solutions.

**FIGURE 4:**
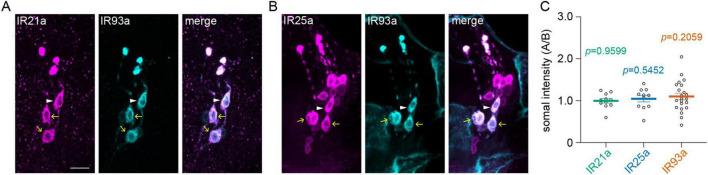
Overexpression of *Ir93a* increases the somal expression of IR93a in B-type DOCCs. **(A,B)** Immunostaining of IR21a [magenta in **(A)**], IR25a [magenta in **(B)**], and IR93a (cyan) in the anterior part of fly larvae. White arrowheads denote B-type DOCCs, and yellow arrows denote A-type DOCCs. Scale bar: 10 μm. **(C)** The somal fluorescence intensities of IR21a, IR25a, and IR93a in A-type to B-type DOCCs. The intensities of two A-type DOCCs within the same DOG were averaged, followed by the calculation of the intensity ratios of A-type over B-type DOCCs. One sample *t*-test or Wilcoxon test, *p*-values are displayed in the figure. Data represent mean ± s.e.m. IR21a: 22 A-type and 11 B-type DOCCs from 6 animals; IR25a: 22 A-type DOCCs and 11 B-type from 6 animals; IR93a: 44 A-type and 22 B-type DOCCs from 12 animals.

The overexpression of *Ir93a* had a selective impact on B-type DOCCs. The detectable B-type DOCCs maintained their temperature responses after extended sucrose exposure (Figures 3I–K) despite the proportion of animals with detectable B-type DOCCs being reduced to the *wt* level (Figure 3L). Unlike in *wt* larvae and those overexpressing *Ir21a* and *Ir25a*, the expressions of IR21a and IR25a were comparable in both A-type and B-type DOCCs, and IR93a had a higher level in A-type than in B-type DOCCs (Supplementary Figures 1A–C and Supplementary Table 1). Overexpressing *Ir93a* eliminated the discrepancy in IR93a expression levels between A-type and B-type DOCCs (Figure 4, Supplementary Figure 1D, and Supplementary Table 1), and restored the detectable B-type calcium responses to cool temperatures after three hours of exposure to sucrose. However, the proportion of B-type DOCCs containing detectable GCaMP baseline was not restored by overexpressing *Ir93a*. Therefore, overexpression of *Ir93a* restores the responses of detectable B-type DOCCs under hypertonic conditions but not their detectability.

## Discussion

In each DOG, three DOCCs are divided into two types according to their distinct dynamics of physiological responses: A-type and B-type DOCCs (Klein et al., 2015). In this study, we further characterized the discrepancies between the two types. We found that prolonged exposure to hypertonic solutions did not affect A-type DOCCs but reduced the proportion of B-type DOCCs containing a detectable GCaMP baseline and diminished the temperature-induced increase in GCaMP fluorescence in those B-type cells that remained detectable. Moreover, B-type DOCCs expressed a lower level of IR93a than A-type DOCCs. Overexpression of *Ir93a* restored the temperature responses of detectable B-type DOCCs under hypertonic conditions but not the proportion of B-type cells with a detectable GCaMP baseline. These findings suggest that B-type DOCCs serve as integrators for thermal and tonic information, with IR93a playing a role in modulating this function.

Tonicity is the ability of a solution to change the volume of cells by altering their water content. A hypotonic solution causes water influx into a cell, while a hypertonic solution induces water efflux from the cell (Maldonado and Mohiuddin, 2023). Disturbances in water homeostasis often cause severe health issues. The two primary etiologies of hypertonicity-related diseases are hyperglycemia and hypernatremia (Rondon-Berrios et al., 2017). For small ectotherms, like *C. elegans*, hypertonic stress can be induced by increasing the osmolarity of their environments, such as by exposing animals to higher concentrations of NaCl or sucrose (Urso and Lamitina, 2021; Lamitina et al., 2004). In *C. elegans*, the hypertonic avoidance behavior relies on the OSM-9 and OCR-2 complex in ASH sensory neurons (Colbert et al., 1997; Bargmann et al., 1990; Tobin et al., 2002). This complex also acts as a temperature receptor (Ohnishi et al., 2020), suggesting that OSM-9 and OCR-2 are responsible for sensing both temperature and tonic stimuli. As in *C. elegans*, fly larvae rely on the same molecular mechanisms to sense these two environmental stimuli. Our findings indicate that fly larvae depend on IRs in B-type DOCCs to sense and integrate temperature and tonic stimuli.

In this study, we tested two hypertonic solutions: sucrose and NaCl solutions. Neither 20% sucrose nor 0.5 M NaCl affected A-type DOCCs, but both significantly reduced the proportion of B-type DOCCs containing a detectable GCaMP baseline and the temperature responses of those that remained detectable with prolonged incubation (Figure 1), suggesting hypertonic environments selectively modulate B-type DOCCs. Interestingly, in 0.5 M NaCl, B-type DOCCs exhibited higher responses than A-type DOCCs within the first hour of incubation (Figure 1R). The phenomenon was unique to 0.5 M NaCl and was not present in sucrose solutions. Two possibilities could lead to this phenomenon: a decrease in A-type responses or an increase in B-type responses. However, neither A-type (Mann–Whitney test, *p* = 0.8112) nor B-type (Unpaired *t*-test, *p* = 0.4427) DOCCs exhibited a difference in responses between exposure to 20% sucrose and 0.5 M NaCl. Further study is necessary to understand how 0.5 M NaCl increases the temperature responses of B-type DOCCs during the first hour of incubation and its physiological importance.

In summary, we identify that compared to A-type DOCCs, B-type DOCCs contain a lower level of IR93a, resulting in smaller temperature responses under hypertonic conditions. However, the different expressions of IR93a cannot explain all discrepancies between A-type and B-type DOCCs. For example, with extended incubation in hypertonic solutions, more than half of B-type DOCCs did not exhibit a detectable GCaMP baseline (Figure 1K). This phenomenon was not restored by enhancing the IR93a level (Figure 3L). Moreover, B-type DOCCs took longer to be activated upon cooling (Supplementary Figure 5A; Klein et al., 2015). Overexpression of IRs did not impact their activation dynamics (Supplementary Figures 5B–D). To fully understand the differences in A-type and B-type functions and mechanisms underlying the distinct responses, we need new genetic tools that separately label A-type and B-type DOCCs.

## Data Availability

The original contributions presented in this study are included in this article/Supplementary material, further inquiries can be directed to the corresponding author.

## Supplementary material

Supplementary Table 1 Microscope settings and raw intensity readings for immunostaining of the indicated genotypes, antibodies, cell types, and incubation times in 20% sucrose solutions.
