# Supplementary material for: The impacts of hypertonic conditions on Drosophila larval cool cells
Source: Front Cell Neurosci. 2024 Sep 23;18:1347460. doi: 10.3389/fncel.2024.1347460 (PMC11459462; doi:10.3389/fncel.2024.1347460)
Supplement: Supplementary file 1 [file Table_2.docx]

***Supplementary Material***


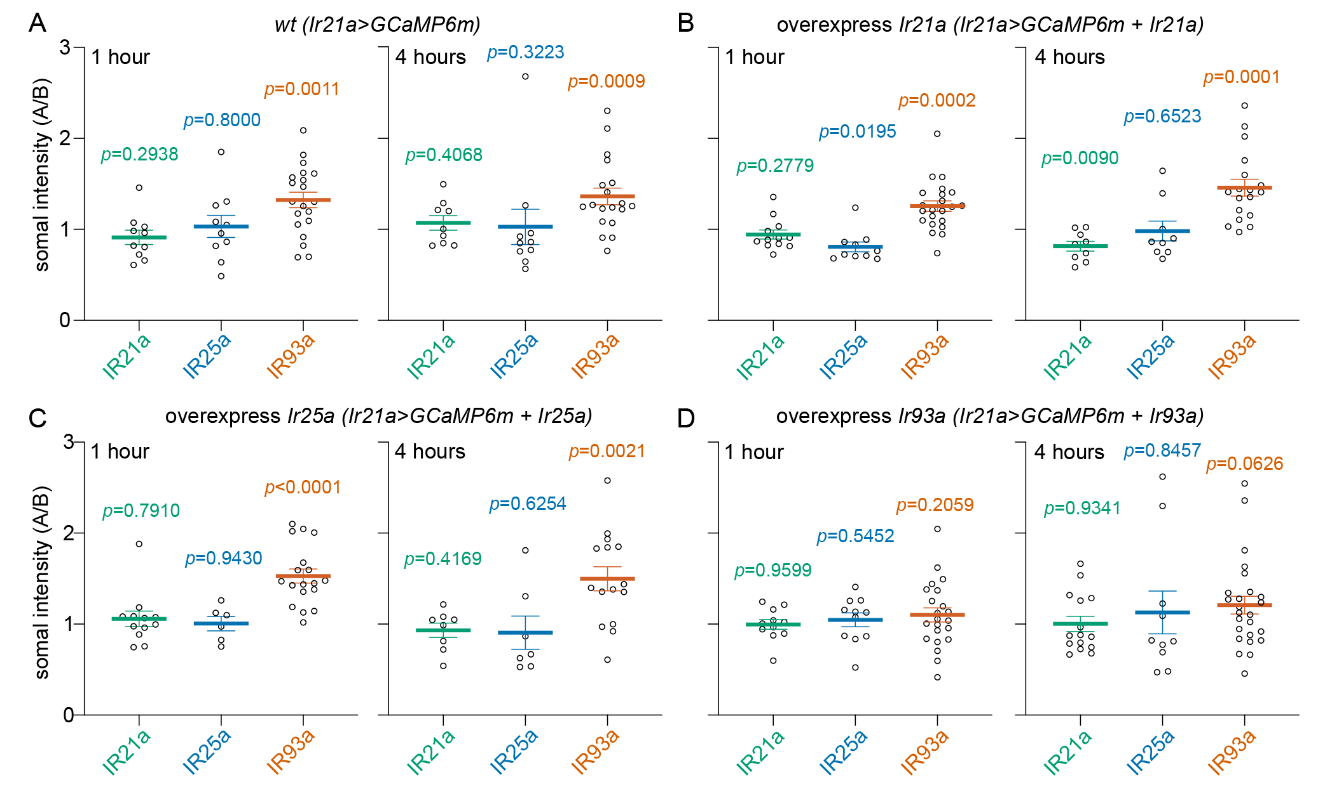


**Supplementary Figure 1.** The impacts of overexpression of *Ir21a, Ir25a,* and *Ir93a* in A-type to B-type DOCCs. The somal fluorescence intensities of IR21a, IR25a, and IR93a in A-type to B-type DOCCs were calculated of indicated genotypes and durations in 20% sucrose. The intensities of two A-type DOCCs within the same DOG were averaged, followed by the calculation of the intensity ratios of A-type over B-type DOCCs. One sample t test or Wilcoxon test, *p* values are displayed in the figure. Data represent mean ± s.e.m. Genotype: *Ir21a>GCaMP6m*: *Ir21a-Gal4/UAS-GCaMP6m* (A); *Ir21a>GCaMP6m + Ir21a*: *Ir21a-Gal4/+;UAS-GCaMP6m/UAS-Ir21a* (B); *Ir21a>GCaMP6m + Ir25a*: *Ir21a-Gal4/UAS-Ir25a;UAS-GCaMP6m/+* (C); *Ir21a>GCaMP6m + Ir93a*: *Ir21a-Gal4/+;UAS-GCaMP6m/UAS-Ir93a* (D). (A) 1 hour: IR21a: 20 A-type and 10 B-type DOCCs from 5 animals; IR25a: 20 A-type and 10 B-type DOCCs from 5 animals; IR93a: 40 A-type and 20 B-type DOCCs from 10 animals (the same data as Fig 2C). 4 hours: IR21a: 18 A-type and 9 B-type DOCCs from 5 animals; IR25a: 20 A-type and 10 B-type DOCCs from 5 animals; IR93a: *n =* 38 A-type DOCCs from 10 animals. (B) 1 hour: IR21a: 24 A-type and 12 B-type DOCCs from 6 animals; IR25a: 20 A-type and 10 B-type DOCCs from 5 animals; IR93a: 44 A-type and 2 B-type DOCCs from 11 animals. 4 hours: IR21a: 18 A-type and 9 B-type DOCCs from 5 animals; IR25a: 18 A-type and 9 B-type DOCCs from 7 animals; IR93a: 36 A-type and 18 B-type DOCCs from 12 animals. (C) 1 hour: IR21a: 24 A-type and 12 B-type DOCCs from 6 animals; IR25a: 12 A-type and 6 B-type DOCCs from 5 animals; IR93a: 36 A-type and 18 B-type DOCCs from 11 animals. 4 hours: IR21a: 16 A-type and 8 B-type DOCCs from 5 animals; IR25a: 14 A-type and 7 B-type DOCCs from 4 animals; IR93a: 30 A-type and 15 B-type DOCCs from 10 animals. (D) 1 hour: IR21a: 22 A-type and 11 B-type DOCCs from 6 animals; IR25a: 22 A-type and 11 B-type DOCCs from 6 animals; IR93a: 44 A-type and 22 B-type DOCCs from 12 animals (the same data from Fig 4C). 4 hours: IR21a: 30 A-type and 15 B-type DOCCs from 8 animals; IR25a: 20 A-type and 10 B-type DOCCs from 5 animals; IR93a: 50 A-type and 25 B-type DOCCs from 13 animals.


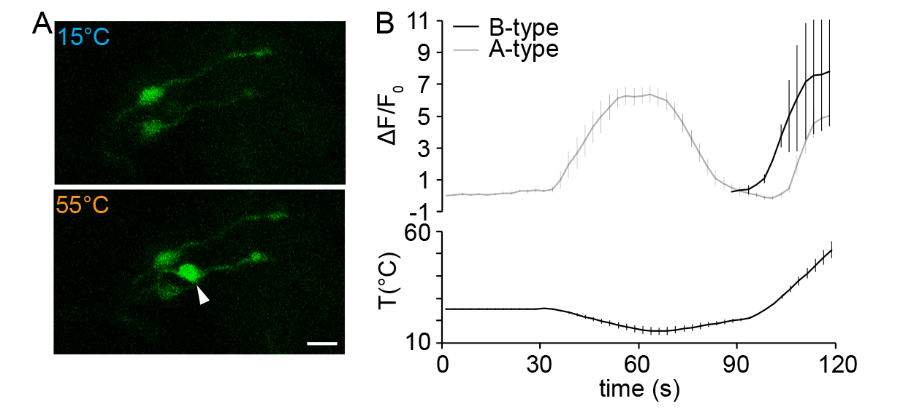


**Supplementary Figure 2.** TRPA1 activates undetectable B-type DOCCs after 3-hour incubation in 20% sucrose. (A) Temperature responses of TRPA1-expressing DOCCs following exposure to 20% sucrose for three hours. Genotype: *Ir21a-Gal4/UAS-TrpA1;UAS-GCaMP6m/+*. White arrowheads denote B-type DOCCs. Scale bar: 10 μm. (B) Calcium changes in response to temperature fluctuations in indicated DOCCs. Fluorescence is quantified as the ratio of fluorescence intensity at the indicated time points to the initial intensity. Data represent mean ± s.e.m.


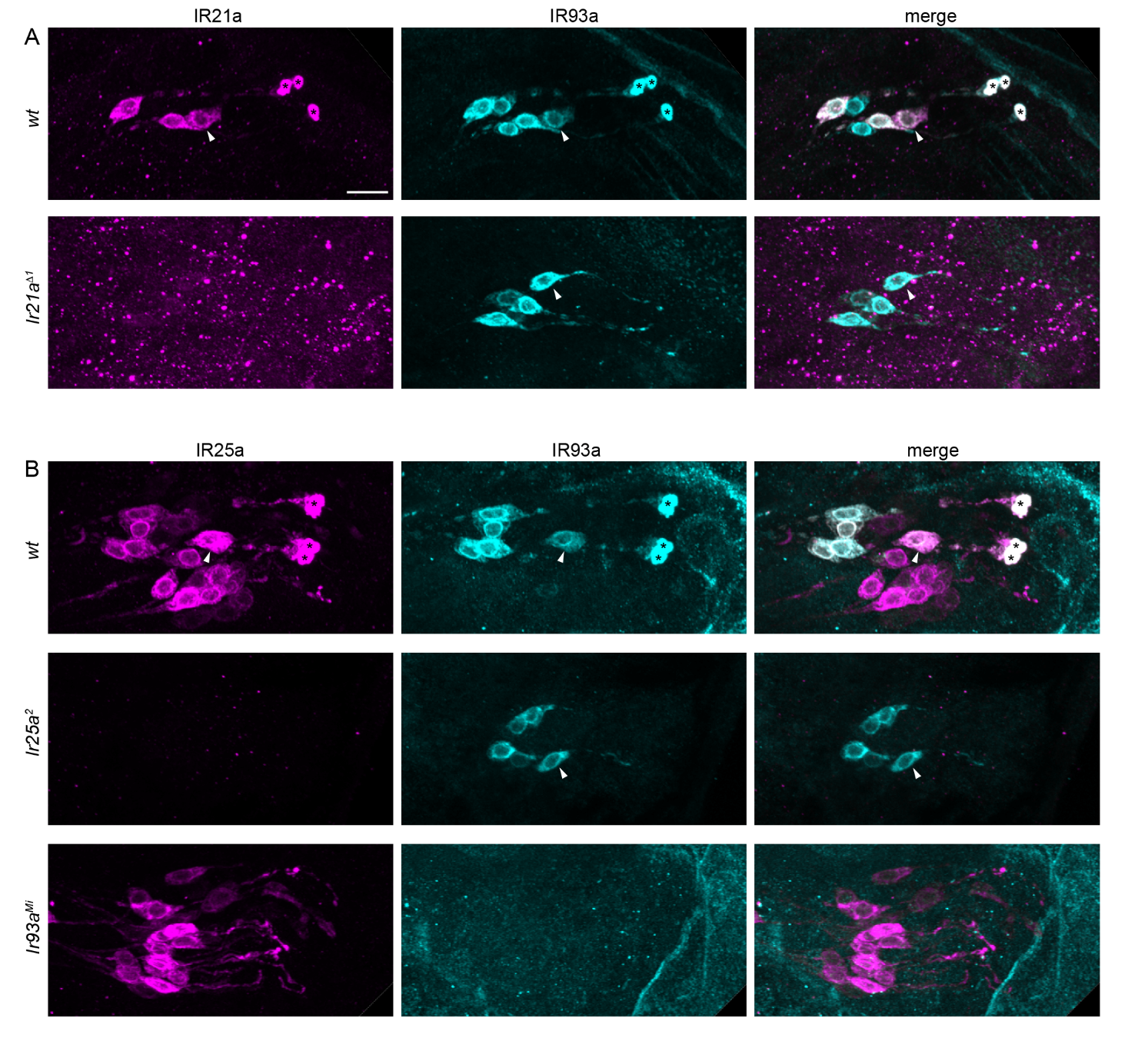
**Supplementary Figure 3.** IR21a, IR25a, and IR93a are not detectable in *Ir21a^Δ1^, Ir25a^2^, and Ir93a^Mi^* mutants. (A) Immunostaining of IR21a (magenta) and IR93a (cyan) in the anterior part of *wt* and *Ir21a^Δ1^* fly larvae. (B) Immunostaining of IR25a (magenta) and IR93a (cyan) in the anterior part of *wt, Ir25a^2^*, and *Ir93a^Mi^* fly larvae. White arrowheads denote B-type DOCCs; asterisks denote dendritic bulbs of DOCCs. Note that *Ir* mutants do not exhibit dendritic bulbs. Scale bar: 10 μm.


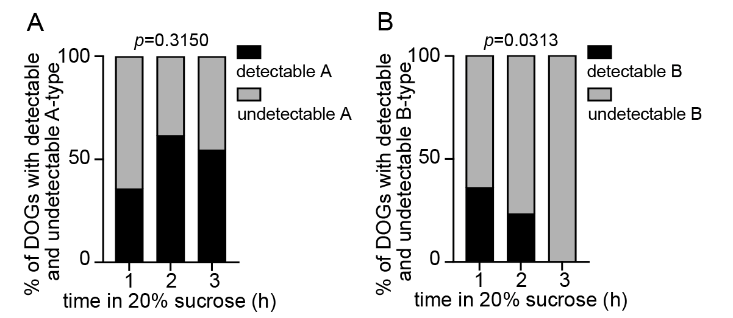


**Supplementary Figure 4.** Overexpression of *Ir25a* decreases the proportion of DOGs containing the detectable A-type and B-type DOCCs. The percentage of DOGs containing detectable A-type (A) and B-type (B) DOCCs of the indicated durations in 20% sucrose. (A) The percentage of detectable A-type DOCCs remains with prolonged incubation in 20% sucrose. (B) The percentage of detectable B-type DOCCs decreases with prolonged incubation in 20% sucrose. Chi-square test for trend, *p* values are displayed in the figure.

**
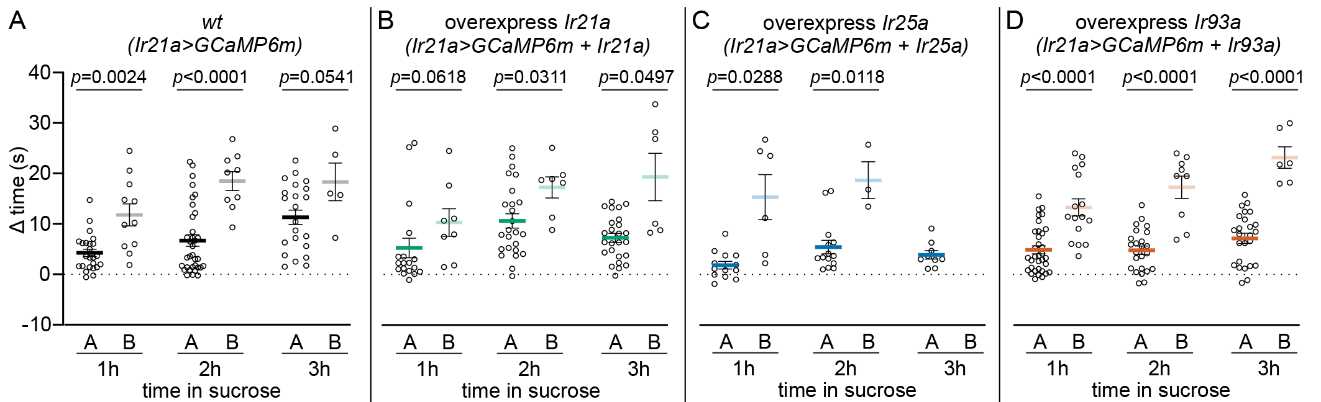
**

**Supplementary Figure 5.** Overexpression of *Ir21a*, *Ir25a*, and *Ir93a* has no effects on activation time of cooling responses. B-type DOCCs exhibit delayed responses compared to A-type DOCCs. Mann-Whitney test or unpaired t test, *p* values are displayed in the figure. Data represent mean ± s.e.m. Genotype: *Ir21a>GCaMP6m*: *Ir21a-Gal4/UAS-GCaMP6m* (A); *Ir21a>GCaMP6m + Ir21a*: *Ir21a-Gal4/+;UAS-GCaMP6m/UAS-Ir21a* (B); *Ir21a>GCaMP6m + Ir25a*: *Ir21a-Gal4/UAS-Ir25a;UAS-GCaMP6m/+* (C); *Ir21a>GCaMP6m + Ir93a*: *Ir21a-Gal4/+;UAS-GCaMP6m/UAS-Ir93a* (D). (A) 1h: 24 A-type DOCCs and 11 B-type DOCCs from 12 DOGs; 2h: 34 A-type DOCCs and 9 B-type DOCCs from 17 DOGs; 3h: 22 A-type DOCCs and 5 B-type DOCCs from 11 DOGs. (B) 1h: 18 A-type DOCCs and 8 B-type DOCCs from 9 DOGs; 2h: 25 A-type DOCCs and 7 B-type DOCCs from 13 DOGs; 3h: 25 A-type DOCCs and 6 B-type DOCCs from 13 DOGs. (C) 1h: 13 A-type DOCCs and 6 B-type DOCCs from 7 DOGs; 2h: 14 A-type DOCCs and 3 B-type DOCCs from 7 DOGs; 3h: 9 A-type DOCCs and 0 B-type DOCCs from 5 DOGs. (D) 1h: 34 A-type DOCCs and 16 B-type DOCCs from 17 DOGs; 2h: 24 A-type DOCCs and 9 B-type DOCCs from 12 DOGs; 3h: 25 A-type DOCCs and 6 B-type DOCCs from 13 DOGs.
